# Social Relationships and Alcohol Abuse in Older Ages

**DOI:** 10.1093/geroni/igaa057.1302

**Published:** 2020-12-16

**Authors:** Eric Vogelsang

**Affiliations:** Cal State University-San Bernardino, San Bernardino, California, United States

## Abstract

In an aging world, researchers and practitioners often extol the health benefits of social relationships (e.g., family ties, social participation) for older adults. Yet, they generally ignore how these same bonds and activities may contribute to negative health behaviors, such as alcohol use and abuse. Using data from the Wisconsin Longitudinal Study (16,065 observations from a cohort of 7,007 respondents), I examine how family structure, family history, and participating in certain social activities predict alcohol consumption and symptoms of alcoholism between ages 53 and 71. I find that having children and taking part in two particular social activities (meeting friends and group exercise) are associated with problematic drinking behavior. Moreover, religious participation and ever living with alcoholics were associated with reporting negative alcohol consequences, but not with alcohol consumption itself. These findings contextualize the increasing rates of alcohol abuse among older adults.

